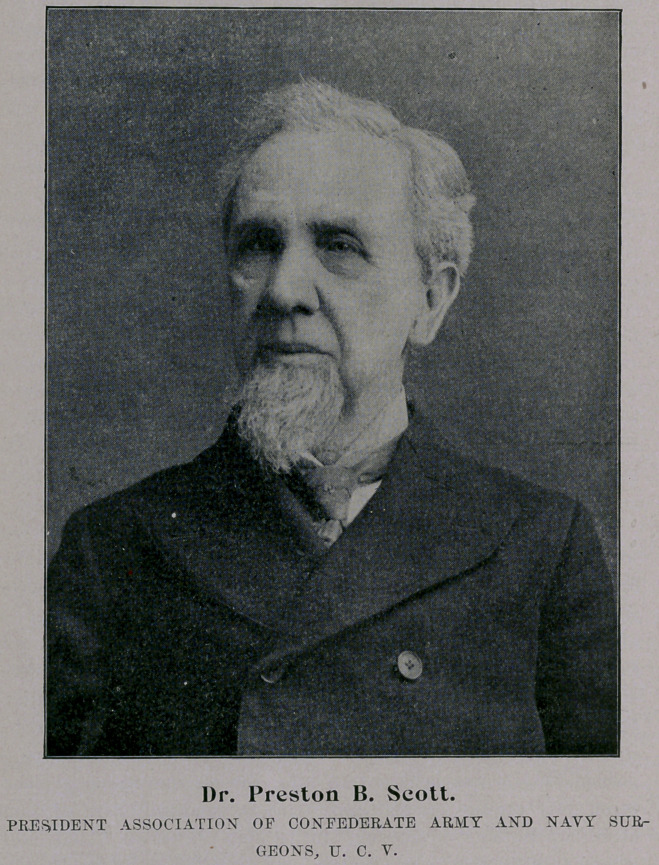# Dr. Preston B. Scott

**Published:** 1900-08

**Authors:** 


**Dr. Preston B. Scott.**

PRESIDENT ASSOCIATION OF CONFEDERATE ARMY AND NAVY SURGEONS, U. C. V.

---

PRESTON B. SCOTT, A. M., M. D., was born in Frankfort, Ky., September 12, 1832. He entered the Confederate service in 1862; was commissioned Surgeon May 1st, that year, and was assigned to the Fourth Kentucky Infantry,—the "Orphan Brigade." He was appointed Associate Medical Director at Jackson, Miss., July, 1863, on staff of Gen. Joseph E. Johnston; October, 1863, was appointed on staff of Lieutenant-General Leonidas Polk, and immediately after the death of Gen. Polk was assigned to duty as Medical Director of the Department of Alabama, Mississippi and East Louisiana, on the Staff of Gen. Stephen D. Lee, and subsequently with Gen. Dick Taylor, commanding that department, and served with him until the close of the war as Medical Director, Field and Hospital. After the war was ended, Dr. Scott returned to Louisville, where he has continuously resided to the present time. At the big reunion of Confederate veterans, held at Louisville last June, under the auspicies of the United Confederate Veterans Association, the Association of Confederate Army and Navy Surgeons was organized and Dr. Scott was unanimously chosen its first president.

## Figures and Tables

**Figure f1:**